# Short-Term Beat-to-Beat QT Variability Appears Influenced More Strongly by Recording Quality Than by Beat-to-Beat RR Variability

**DOI:** 10.3389/fphys.2022.863873

**Published:** 2022-04-01

**Authors:** Ondřej Toman, Katerina Hnatkova, Martina Šišáková, Peter Smetana, Katharina M. Huster, Petra Barthel, Tomáš Novotný, Irena Andršová, Georg Schmidt, Marek Malik

**Affiliations:** ^1^Department of Internal Medicine and Cardiology, University Hospital Brno, Brno, Czechia; ^2^Department of Internal Medicine and Cardiology, Faculty of Medicine, Masaryk University, Brno, Czechia; ^3^National Heart and Lung Institute, Imperial College, London, United Kingdom; ^4^Wilhelminenspital der Stadt Wien, Vienna, Austria; ^5^Klinikum rechts der Isar, Technische Universität München, Munich, Germany

**Keywords:** healthy volunteers, long-term ECG, short-term ECG measurements, QT variability, RR variability, ECG noise contents, immediate RR interval effect, regression-based correction

## Abstract

Increases in beat-to-beat variability of electrocardiographic QT interval duration have repeatedly been associated with increased risk of cardiovascular events and complications. The measurements of QT variability are frequently normalized for the underlying RR interval variability. Such normalization supports the concept of the so-called immediate RR effect which relates each QT interval to the preceding RR interval. The validity of this concept was investigated in the present study together with the analysis of the influence of electrocardiographic morphological stability on QT variability measurements. The analyses involved QT and RR measurements in 6,114,562 individual beats of 642,708 separate 10-s ECG samples recorded in 523 healthy volunteers (259 females). Only beats with high morphology correlation (*r* > 0.99) with representative waveforms of the 10-s ECG samples were analyzed, assuring that only good quality recordings were included. In addition to these high correlations, SDs of the ECG signal difference between representative waveforms and individual beats expressed morphological instability and ECG noise. In the intra-subject analyses of both individual beats and of 10-s averages, QT interval variability was substantially more strongly related to the ECG noise than to the underlying RR variability. In approximately one-third of the analyzed ECG beats, the prolongation or shortening of the preceding RR interval was followed by the opposite change of the QT interval. In linear regression analyses, underlying RR variability within each 10-s ECG sample explained only 5.7 and 11.1% of QT interval variability in females and males, respectively. On the contrary, the underlying ECG noise contents of the 10-s samples explained 56.5 and 60.1% of the QT interval variability in females and males, respectively. The study concludes that the concept of stable and uniform immediate RR interval effect on the duration of subsequent QT interval duration is highly questionable. Even if only stable beat-to-beat measurements of QT interval are used, the QT interval variability is still substantially influenced by morphological variability and noise pollution of the source ECG recordings. Even when good quality recordings are used, noise contents of the electrocardiograms should be objectively examined in future studies of QT interval variability.

## Introduction

The temporal dynamics of ventricular myocardial repolarisation are of interest since increases in beat-to-repolarisation variability have been reported to signify an increased risk of arrhythmic complications and cardiovascular death (Baumert et al., 2016; Hasan and Abbott, 2016). While other possibilities of electrocardiographic measurements have been published (Hasan et al., 2012; Schmidt et al., 2016; Rahola et al., 2021), most frequent expressions of ventricular repolarisation variability are based on beat-to-beat changes in QT interval duration. Risk prediction based on QT variability and its diagnostic utility has been reported in patients with ischaemic heart disease (Hasan et al., 2013), in cardiomyopathy patients (Fischer et al., 2015; Orosz et al., 2015a), in patients with long QT syndrome (Porta et al., 2015; Seethala et al., 2015), in recipients of automatic implantable cardioverter-defibrillators (Monasterio et al., 2013; Smoczyńska et al., 2020) as well as in a variety of other clinically and pathologically defined conditions (Orosz et al., 2015b; Viigimae et al., 2017; Nussinovitch et al., 2018).

Frequently, QT variability is expressed by the so-called QT variability index that was proposed by Berger et al. (1997) in the seminal publication on the topic of QT variability. This index, as repeatedly reviewed (Dobson et al., 2013), relates the beat-to-beat variance of QT interval durations to the simultaneously measured variance of RR interval durations.

In principle, the QT variability index aims at normalizing the beat-to-beat changes of QT interval duration for the underlying changes in the RR intervals, reflecting approximately the concept that QT interval duration needs to be corrected (or normalized) for the duration of the preceding RR interval [i.e., the concept of the so-called immediate RR interval effect (Fossa et al., 2002)]. The QT variability index is therefore a combined measure; its increases may be caused both by increases in the QT variance and by decreases of RR variance, both of which are known indicators of increased risk of cardiovascular events and complications (Malik and Camm, 1990; ESC/NASPE Task Force, 1996; Baumert et al., 2016).

QT interval variability without any correction for the RR variability has also been researched (Niemeijer et al., 2014; Baumert et al., 2016; van den Berg et al., 2019) including the reports of normal values in short-term ECG. While such studies might still have included data influenced by the underlying RR variability, physiologically important technical studies were also reported, investigating the QT interval variability with RR interval effects removed by regression modeling (Porta et al., 2010, 2020; El-Hamad et al., 2019). These studies have been based on shorter ECG recordings that allowed QT interval measurements on every beat basis.

Our experience suggests that in long-term ECG recordings, beat-to-beat QT interval measurements might be influenced by the quality of the signals, even if the measurement is performed only in carefully selected segments of the recordings. We have therefore conducted a study that compared the influence of underlying RR variability and of signal quality on beat-to-beat QT interval variability in segments extracted from long-term 12-lead Holter recordings obtained in a large population of normal healthy volunteers who were investigated during clinical pharmacology studies.

## Methods

### Investigated Population and Electrocardiographic Recordings

Clinical pharmacology studies enrolled 523 healthy volunteers including 259 females, with no statistical age differences between females and males (33.4 ± 9.1 vs. 33.7 ± 7.8 years). Before study enrollment, all the volunteers had a normal standard clinical ECG and normal clinical investigation. The studies were conducted at 3 different clinical research sites that used the same standard inclusion and exclusion criteria mandated for Phase I pharmacology studies (ICH Guideline, 2001), including negative recreational substances tests and negative pregnancy tests for females. All the source studies were ethically approved by the institutional ethics bodies (Parexel in Baltimore; California Clinical Trials in Glendale; and Spaulding in Milwaukee) and all subjects gave informed written consent to study participation and to the scientific investigation of data collected during the studies. The population used in this investigation was the same as reported in a previous study in which we reported the influence of underlying heart rate on QT interval variability (Andršová et al., 2020). However, for the purposes of this investigation, we applied different data processing techniques.

In each study participant, repeated three to four long-term 12-lead Holter ECG recordings with Mason-Likar electrode positions were obtained covering the full day-time periods, i.e., ~14-h periods during which the subjects were not allowed to sleep [to eliminate the influence of sleep on the QT interval duration (Browne et al., 1983; Lanfranchi et al., 2002; Viigimae et al., 2015)]. During these periods, the subjects were not allowed to smoke and/or consume alcohol or caffeinated drinks. No medication was administered during the day of the analyzed Holter recordings and if any medication was administered previously, the recordings were made after appropriate wash-out gaps. The protocols of the different studies were also mutually consistent in respect of the clinical conduct during the drug-free baseline days. Thus, only drug-free data are presented here making further details of source pharmacology studies irrelevant.

Using previously described methods (Malik et al., 2008a, 2012a), multiple non-overlapping and non-adjacent 10-s ECG segments were extracted from the long-term ECGs. The segments were selected with the aim of capturing different heart rates available in the Holter recording. All the extracted 10-s segments contained only sinus rhythm recordings and were free of any ectopic beats. In more detail, the selected segments were extracted (a) from pre-specific time-points of the source pharmacologic studies, (b) from scans of the recordings aimed at finding a representative spectrum of different underlying heart rates of selected ECG segments that were not preceded by heart rate changes exceeding ±2 beat per minute, and (c) from scans of the recordings aimed at finding representative spectrum of heart rate changes preceding the selected ECG segments. A minimum 20-s gap between selected segments was maintained. The ECG segments were selected only if a satisfactory algorithmic measurement of QT interval was possible (Malik et al., 2008a, 2012a).

In each of these ECG segments, QT interval was measured following published procedures (Malik et al., 2008a, 2012a) that included repeated visual controls of all the measurements. Consistency of the interpretation of corresponding ECG morphologies was also assured (Hnatkova et al., 2009). The visually verified QT interval measurements were made in the representative median waveforms of the 10-s segments (sampled at 1,000 Hz) with the superimposition of all 12 leads on the same isoelectric axis (Malik, 2004; Xue, 2009). For each ECG segment, computerized QT interval measurement in the representative median waveform was visually verified and, where necessary, manually corrected by two independently working cardiologists. In case of their disagreement, the final measurement decision was made by a senior member of the team. All visual decisions included the possibility of excluding an ECG segment if the QT interval measurement was considered not reliable (e.g., because of low voltage T waves or because of visible noise pollution that interfered with the measurement).

### Beat-to-Beat QT Interval Measurements

Using a previously proposed technique (Berger et al., 1997; Baumert et al., 2008), QT interval was projected to individual beats within the 10-s ECG by finding the maximum correlation between the representative waveform (in which the original measurement was made) and the signal of the individual QRS-T complexes. The maximum correlations were identified separately for the surroundings of the QRS onset and of the T wave offset. Since it has previously been observed that this process might lead to slightly different results when applied to different ECG leads (Malik, 2008), the cross-correlation technique was applied to the vector magnitude of algebraically reconstructed orthogonal leads (Guldenring et al., 2012).

After the cross-correlation techniques were applied and the corresponding positions of QRS onset and T wave offset were identified in each beat of the analyzed 10-s ECG segment, Pearson correlation coefficients were calculated between the analyzed beat and representative median waveform in windows of ±40 ms surrounding the QRS onset and ±50 ms surrounding the T wave offset. The QT interval measurement on the given beat was accepted only if both these correlation coefficients exceeded 0.99. This assured that substantially noisy beats were excluded.

A 10-s ECG segment in which the QT measurement of the representative waveform was previously performed was accepted for the data analysis of this study if it contained at least two beats with accepted QT interval measurements.

For each beat with accepted QT interval measurement (i.e., an accepted beat in an accepted 10-s segment), the duration of the preceding RR interval was obtained. For each 10-s segment, the median duration of the accepted QT interval measurements and the median duration of the accepted preceding RR intervals were obtained. The heart rate of the 10-s ECG segment was also obtained from all RR intervals regardless of whether the subsequent QT interval measurements were accepted.

### ECG Noise and Morphological Variability

Although the strict acceptance limit imposed on the cross-correlation coefficients between the individual beats and the representative median waveform assured that beats substantially polluted by noise were eliminated from the analysis, it was still proper to investigate the morphological differences between the individual beats and the 10-s representative waveform.

For this purpose, in each accepted beat, the SD of the voltage differences between the representative waveform and the individual leads was calculated, for each ECG lead, in the windows of ±100 ms surrounding the aligned QRS onsets and T wave offset (possible overlaps between subsequent beats during fast heart rate were eliminated). In each ECG lead and each beat, the average of these two standard deviations of voltage differences was calculated and the median value of these measurements across all 12 ECG leads represented the morphological departure of the measured beat from the 10-s representative waveform. Although these signal differences might have been caused by biologically determined signal variability [e.g., respiration induced (Noriega et al., 2012)] we shall use the term “beat noise” for the purpose of this investigation.

In each 10-s ECG segment, the median value of the beat noise, termed here the “10-s median beat noise,” was calculated over all accepted beats and used to characterize the morphological stability of the complete 10-s segment.

### Individual Beat Analysis

For each accepted beat, the difference ΔQT between the measured QT interval and the median of accepted QT interval durations in the 10-s ECG segment was calculated and correspondingly, the difference ΔRR was obtained between the RR interval preceding an accepted beat and the median of RR interval preceding accepted beats in the same 10-s ECG segment.

The concept of the “immediate RR interval effect” postulates that a longer RR interval should be followed by a longer QT interval. To assess the validity of this concept, we pooled, in each study subject separately, all the ΔQT and ΔRR pairs from all accepted beats in all processed 10-s ECG segments.

Three different analyses of the ΔQT and ΔRR pairs were performed: Firstly, in each study subject, a linear regression between ΔQT and ΔRR was calculated. Secondly, the data ΔQT and ΔRR pair was called concordant if either (ΔRR > 0 and ΔQT > 0) or (ΔRR < 0 and ΔQT < 0) and likewise, it was called discordant if (ΔRR < 0 and ΔQT > 0) or (ΔRR > 0 and ΔQT < 0). We have calculated the proportions of concordant and discordant ΔQT, ΔRR pairs among all accepted beats (in each study subject separately) and, to eliminate minimal data fluctuations, repeated the calculations of the proportions of concordant and discordant ΔQT, ΔRR pairs considering only those concordant and discordant beats for which the absolute value |ΔRR| exceeded 20 ms, and 50 ms. Thirdly, in each study subject, the Spearman correlation coefficient between |ΔQT| and |ΔRR| pairs were compared with the Spearman correlation coefficient between |ΔQT| values and the beat-wise corresponding values of the beat noise.

### ECG Segment Analysis

In each ECG segment, the SD of ΔQT and ΔRR values was obtained (it is easy to see that these values were identical to the within segments SD of QT and RR interval values—we shall use the abbreviations SDQT and SDRR to denote these within-segment measurements).

The intra-subject averages of SDQT and SDRR values as well as of median beat noise values were compared between female and male study participants, and also related to the subject ages.

Within each subject, Spearman correlations were computed between SDQT, SDRR, and median beat noise obtained from the same ECG segments. Similar Spearman correlations were also calculated between SDQT and heart rate of the ECG segment, and SDQT and the median QT interval duration in the ECG segment. The median QT duration of an ECG segment was calculated using only the accepted beats.

#### Correction of SDQT

To investigate the covariates of SDQT, intra-subject linear regressions were performed. That is, in each subject in whom N accepted 10-s ECG segments were available, values $\{{𝔖}_{i}^{QT}{\}}_{i=1}^{N}$, ${\left\{{𝔖}_{i}^{RR}\right\}}_{i=1}^{N}$, ${\left\{{℧}_{i}\right\}}_{i=1}^{N}$, ${\left\{H_{i}\right\}}_{i=1}^{N}$, and ${\left\{Q_{i}\right\}}_{i=1}^{N}$ of serial measurements of SDQT, SDRR, 10-s median beat noise, 10-s heart rate, and 10-s median QT interval, respectively, were used to construct linear regressions:


(1) $$\begin{array}{l} {𝔖}_{i}^{QT}=\alpha_{1}+\alpha_{2}{𝔖}_{i}^{RR}+\epsilon_{i}^{\left(1\right)} \end{array}$$

(2) $$\begin{array}{l} {𝔖}_{i}^{QT}=\beta_{1}+\beta_{2}{℧}_{i}+\epsilon_{i}^{\left(2\right)} \end{array}$$

(3) $$\begin{array}{l} {𝔖}_{i}^{QT}=\vartheta_{1}+{\vartheta_{21}{𝔖}_{i}^{RR}+\vartheta }_{22}{℧}_{i}+\epsilon_{i}^{\left(3\right)} \end{array}$$

(4) $$\begin{array}{l} {𝔖}_{i}^{QT}=\zeta_{1}+{\zeta_{21}{𝔖}_{i}^{RR}+\zeta }_{22}{℧}_{i}+\zeta_{23}H_{i}+\epsilon_{i}^{\left(4\right)} \end{array}$$

(5) $$\begin{array}{l} {𝔖}_{i}^{QT}=\xi_{1}+{\xi_{21}{𝔖}_{i}^{RR}+\xi }_{22}{℧}_{i}+\xi_{23}H_{i}+\xi_{24}Q_{i}+\;\epsilon_{i}^{\left(5\right)} \end{array}$$

where the regression coefficients were obtained by standard matrix equations to obtain zero centered $\epsilon_{i}^{\left(■\right)}$errors with minimal sums of their squares.

Using these equations, regression intercepts α_1_, β_1_, and ϑ_1_ were interpreted as intra-subject central values of SDQT “corrected” for the influence of SDRR, the influence of 10-s median beat noise, and the combined influence of SDRR and 10-s median beat noise. That is, using the same principles as used in subject-specific heart rate corrections of QT interval and of other interval measurements (Hnatkova et al., 2017) that produce correction values for RR interval of 1 s, the intercepts α_1_, β_1_, and ϑ_1_ were used as estimates of intra-subject SDQT values corrected for zero SDRR, zero beat noise, and combined zero SDRR and zero beat noise. No such correction of SDQT was based on equations (4) and (5).

For all regression equations (1) through (5), the values $\sqrt{\overset{N}{\underset{i=1}{\sum }}{\left(\epsilon_{i}^{\left(■\right)}\right)}^{2}}$, that is the residuals of the equations, were compared with the SD of all SDQT measurements (i.e., SD of all ${𝔖}_{i}^{QT}$ values). This comparison of the residuals allowed us to study the effects of combined co-variates on the intra-subject reproducibility of SDQT. This allowed studying the intra-subject variability of SDQT measurements made in different 10-s ECGs and the reduction of such variability by eliminating the influence of SDRR, of 10-s median beat noise, and of the combinations of SDRR and 10-s median beat noise with 10-s heart rate and 10-s median QT interval.

### Statistics and Data Presentation

Descriptive data are presented as means ± SD. Distributions of Spearman correlation coefficients computed in individual subjects are presented as medians and inter-quartile ranges (IQR). Comparisons between females and males were based on a two-sample two-tail *t*-test assuming different variations between compared datasets and subsequently confirmed by the non-parametric Kolmogorov-Smirnov test. The significance of linear regression slopes between age and the investigated indices was tested using the Fisher–Snedecor F distribution.

The comparisons between the proportions of regression residuals and other intra-subject comparisons used non-parametric paired Wilcoxon test. The calculation of the multivariable linear regressions repeated in different study subjects utilized an in-house matrix manipulation software package programmed in C++. The differences between the measured intra-subject means of SDQT values and the values corrected for zero SDRR and/or for zero beat noise were expressed in relative terms, that is by calculating the proportions of (SDQT–SDQT_*corrected*_)/SDQT, where the SDQT_*corrected*_ is the regression corrected value. We term these proportions the “SDQT reductions” and express them in per-cent. Using the same proportions between intra-subject SDQT variability and the residuals of regressions (1) to (5), we obtained regression-based reductions of intra-subject SDQT variability which we term the “residual reductions” and express them again in per-cent.

Statistical tests used IBM SPSS package, version 27 (IBM, Armonk, New York, USA). *P*-values below 0.05 were considered statistically significant. Because of the interdependence between the different indices, no correction for the multiplicity of statistical testing was made.

## Results

The study was based on a total of 642,708 individual 10-s ECG samples and a total of 6,114,562 individual beats accepted for the analysis. On average, there were 1,208 ± 228 and 1,248 ± 224 ECG segments, and 11,610 ± 2,821 and 11,768 ± 2,831 individual beats processed in female and male subjects, respectively (no statistical differences between sexes).

**Figure 1:**
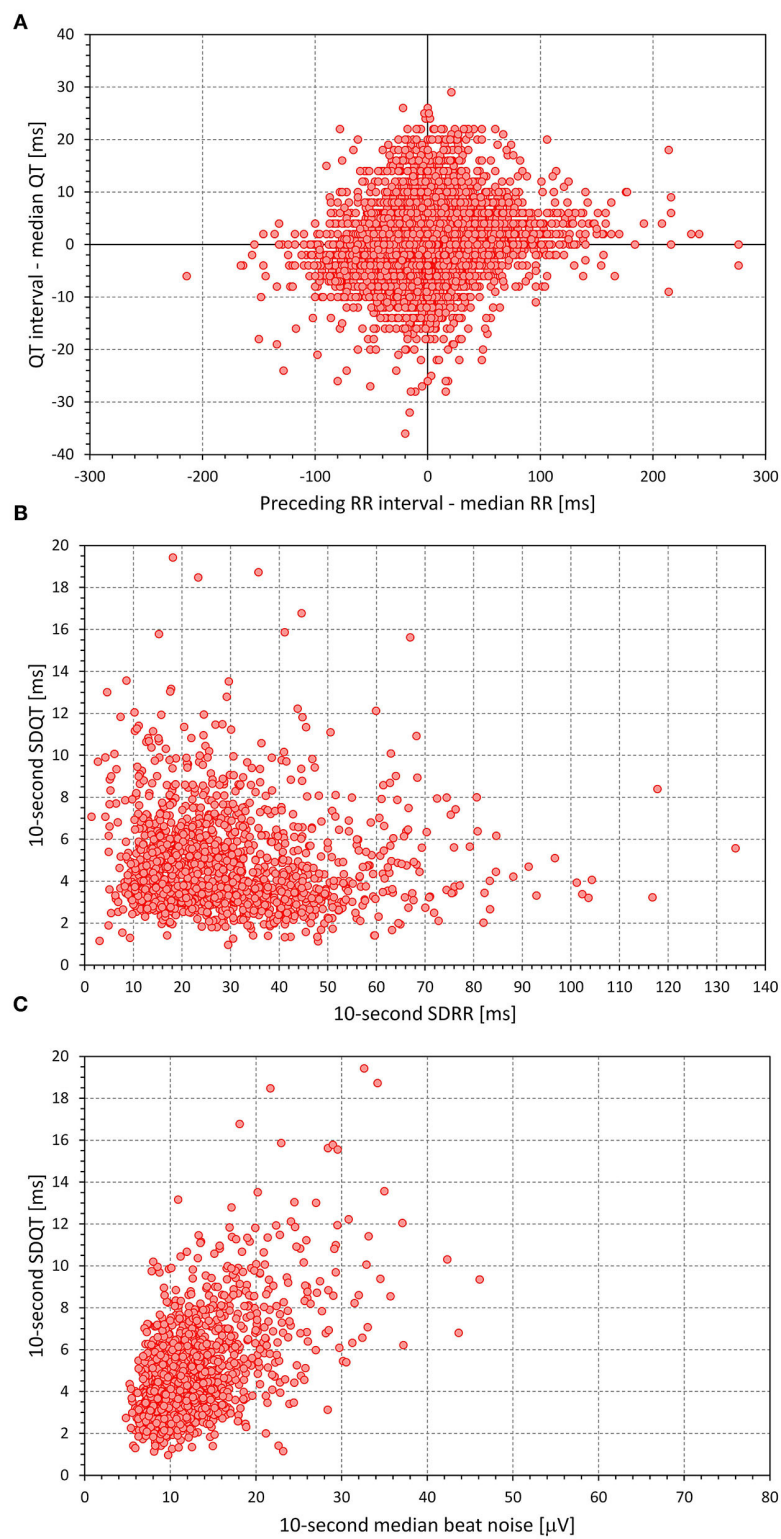
Example of data distribution in a 36-year-old female. The top **(A)** shows a scatter diagram between the ΔRR and the ΔQT value measured in all individual beats accepted in the data analysis of this subject. The middle **(B)** and bottom **(C)** were derived from the data of accepted 10-s ECG segments of the Holter recordings of the subject. The middle **(B)** shows a scatter diagram between SDRR and SDQT values, the bottom **(C)** shows a scatter diagram between median beat noise and the SDQT values. Note the wide spread of the data in all panels and the visible data trend in the bottom panel.

**Figure 2:**
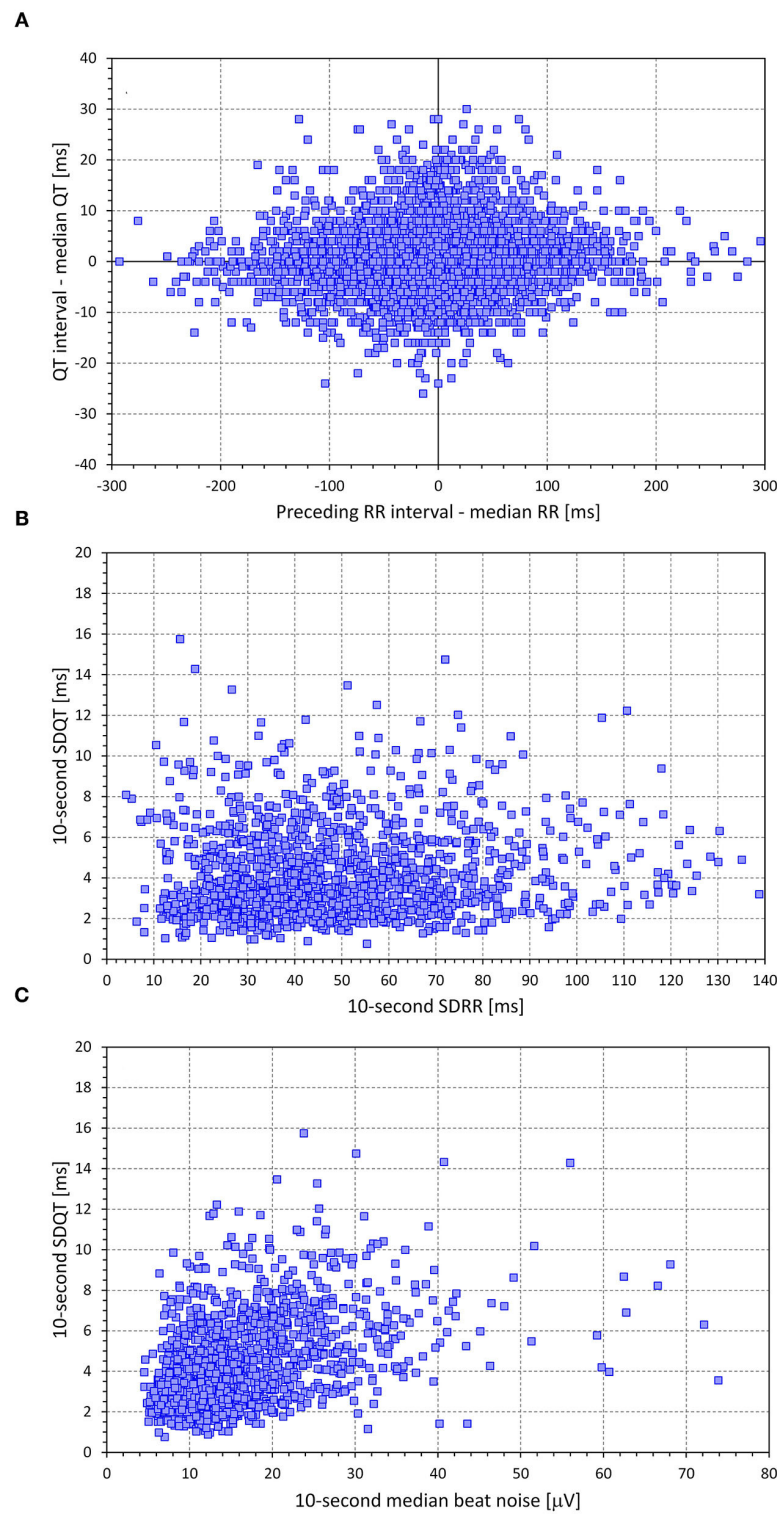
Example of data distribution in a 34-year-old male. The layout of the figure is the same as in Figure 1.

Figures 1, 2 show examples of the distribution and inter-dependency of ECG measurements in two study subjects. The relationships between the measurements seen in the figures appeared visually representative of the data in other subjects.

### Analysis of Individual Beats

#### Immediate RR Interval Effect

**Figure 3:**
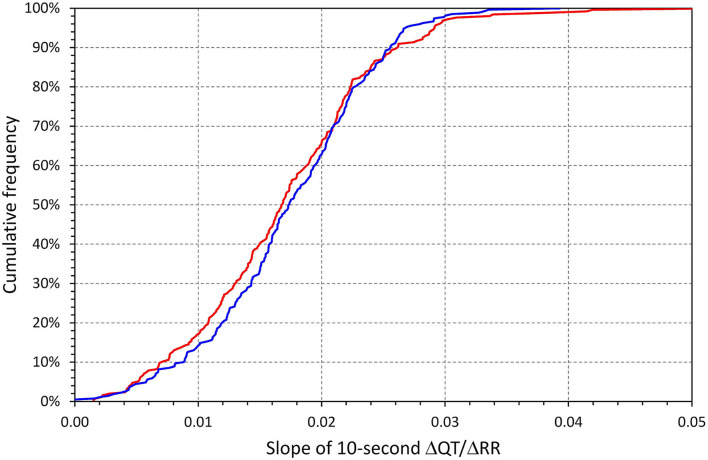
Cumulative distributions of intra-subject linear ΔQT/ΔRR regression slopes. In each subject, the regression involved all individual beats accepted for the given subject. The red and blue lines correspond to females and males, respectively.

Figure 3 shows the distribution of intra-subject linear-regression ΔQT/ΔRR slopes calculated when pooling, in each study subject separately, all individual analyzed beats. These slopes were 0.0171 ± 0.0076 and 0.0174 ± 0.0066 in females and males, respectively (no statistical difference between sexes).

Pooling all accepted beats together, the intra-subject standard deviation of ΔQT was 4.88 ± 1.05 ms in females and 4.52 ± 0.94 ms in males (*p* < 0.0001). This was very similar to the ΔQT/ΔRR regression residuals which were 4.78 ± 1.08 ms in females and 4.42 ± 0.96 ms in males (*p* < 0.0001). Thus, relating ΔQT linearly to ΔRR reduced the intra-subject standard deviations only (median, IQR) by 1.59% (0.80–2.87%) in females and by 1.99% (1.14–3.11%) in males.

#### Concordant and Discordant Beats

**Figure 4:**
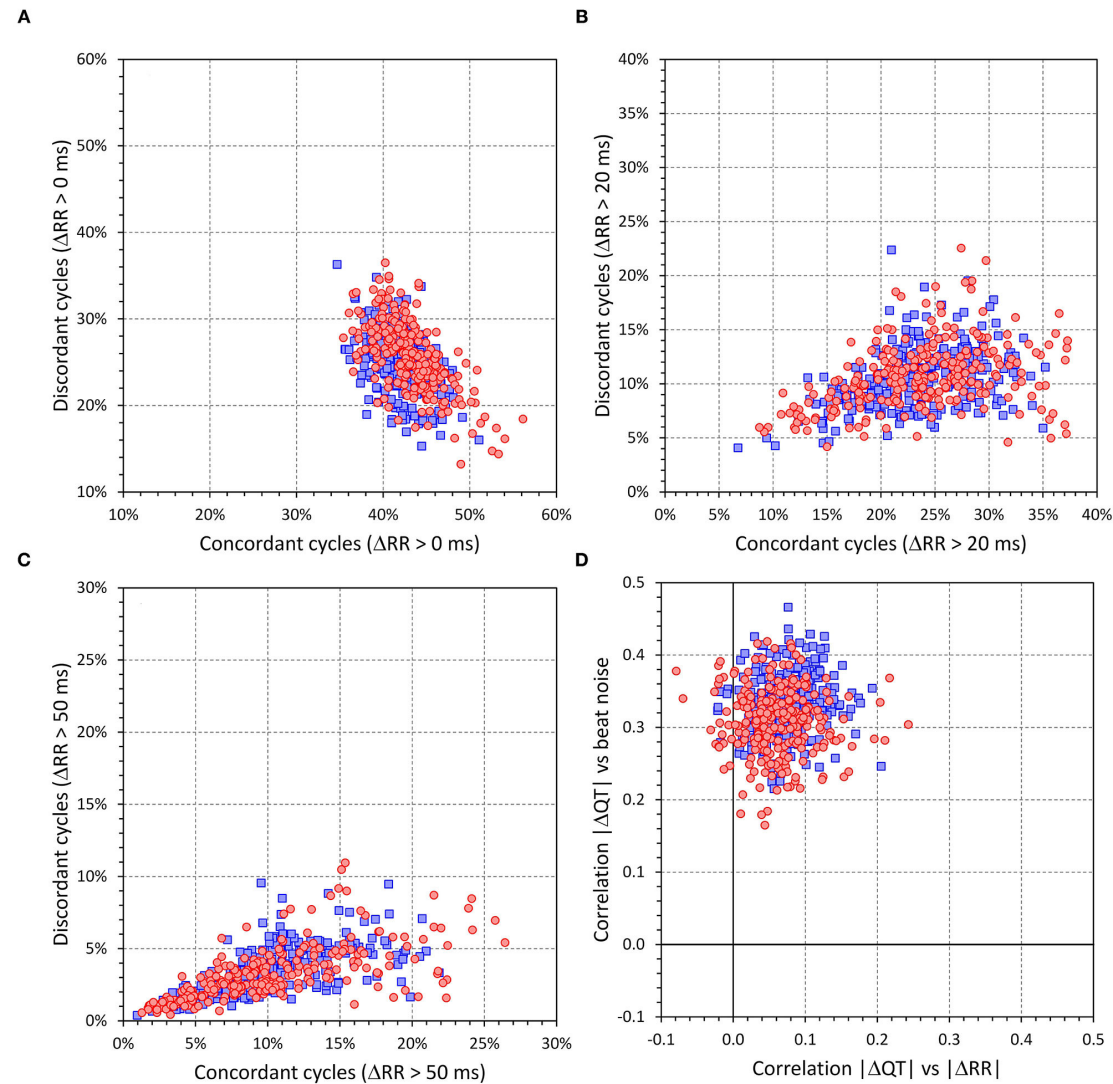
The upper left **(A)** shows a scatter diagram between intra-subject frequencies of concordant and discordant beats (the frequencies calculated as the proportions of all accepted beats for the given subject). The upper right **(C)** and lower left **(B)** show the same for concordant and discordant beat frequencies when considering only the beats for which |ΔRR| > 20 ms and |ΔRR| > 50 ms, respectively. The bottom right **(D)** shows a scatter diagram between intra-subject correlations |ΔQT| vs. |ΔRR| and |ΔQT| vs. beat noise. In all panels, red circles and blue squares correspond to females and males, respectively.

**Figure 5:**
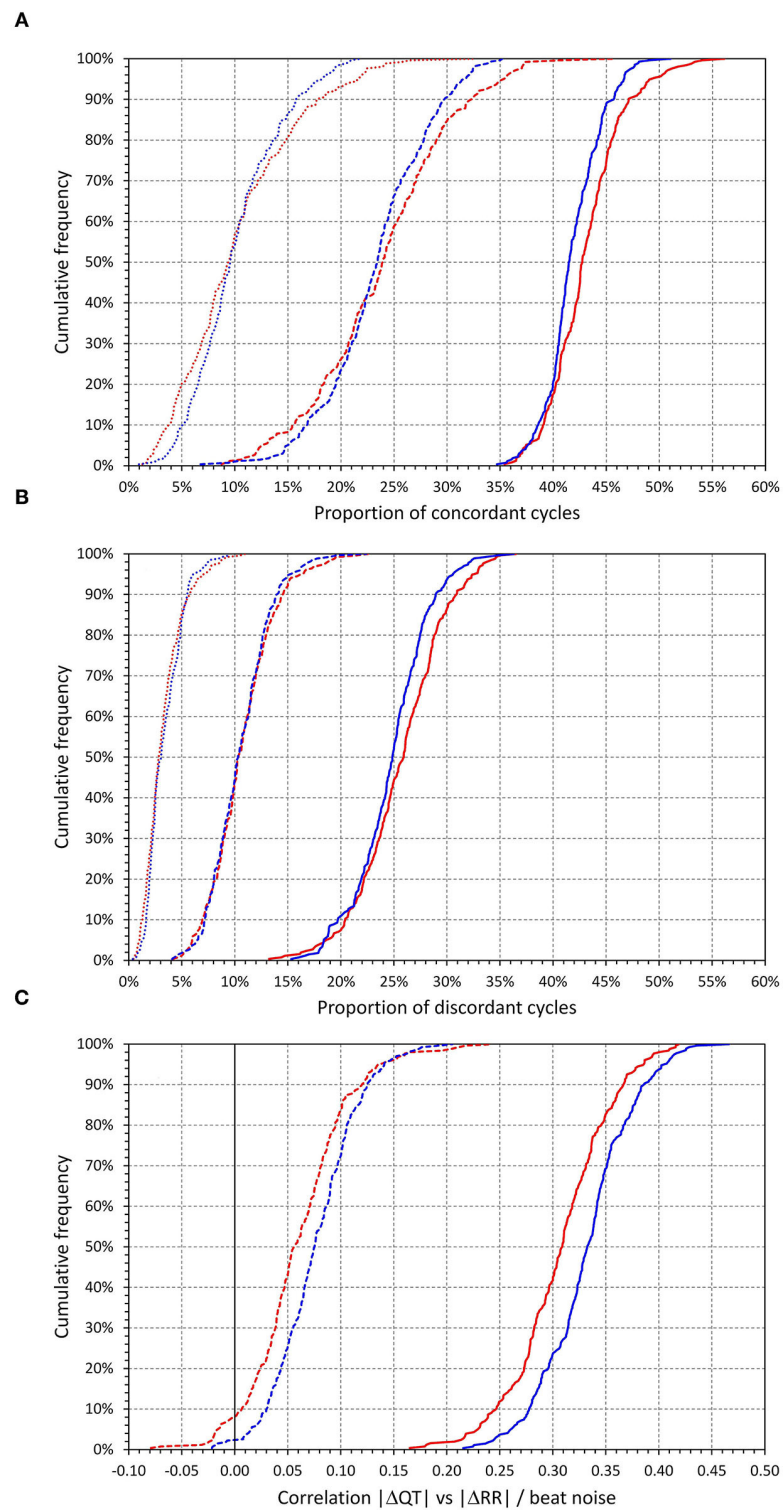
Cumulative distributions of the intra-subject proportions of concordant beats [top **(A)**], the proportions of discordant beats [middle **(B)**], and of the intra-subject correlations between |ΔQT| and |ΔRR|, and between |ΔQT| and beat noise [bottom **(C)**]. In the upper two panels, the solid lines, dashed lines, and dotted lines correspond to all concordant/discordant beats, concordant/discordant beats for which |ΔRR| > 20 ms, and concordant/discordant beats for which |ΔRR| > 50 ms, respectively. In the bottom panel, the solid and dashed lines correspond to intra-subject correlations |ΔQT| vs. beat noise, and intra-subject correlations |ΔQT| vs. |ΔRR|, respectively. In all panels, the red and blue lines correspond to females and males, respectively.

The intra-subject frequencies of concordant and discordant beats are shown in Figure 4. In relation to all beats accepted for analysis, the intra-subject frequencies of concordant beats were 43.1 ± 3.5% and 41.9 ± 2.7% in females and males (*p* < 0.0001), the frequencies of discordant beats were 25.7 ± 4.1% and 24.8 ± 3.5% in females and males (*p* = 0.008). When considering only beats with |ΔRR| > 20 ms, the frequencies of concordant and discordant beats were 23.6 ± 5.7 and 10.6 ± 3.0%, respectively (no sex difference); with |ΔRR| > 50 ms, the corresponding frequencies were 10.2 ± 4.9 and 3.3 ± 1.8%, respectively (no sex difference). Figure 5 presents cumulative distributions of the data shown in Figure 4.

#### Correlations

The intra-subject correlations between |ΔRR| and |ΔQT|, and between beat noise and |ΔQT| are also shown in Figure 4. The |ΔQT| vs. |ΔRR| correlation coefficients were (median and IQR) 0.056 (0.034–0.087) and 0.075 (0.050–0.102) in females and males, respectively (*p* < 0.0001 for the sex differences). In 53 females (20.9%) and 27 males (10.0%), the correlation between |ΔQT| vs. |ΔRR| was not significantly positive. The |ΔQT| vs. beat noise correlation coefficients were 0.309 (0.278–0.336) and 0.333 (0.306–0.355) in females and males, respectively (*p* < 0.0001 for the sex differences). The |ΔQT| vs. beat noise correlation was stronger than the |ΔQT| vs. |ΔRR| correlation in every study subject. See also Figure 5 for the cumulative distributions of these correlation data.

### Analysis of 10-S ECG Segments

#### Sex Comparisons

**Figure 6:**
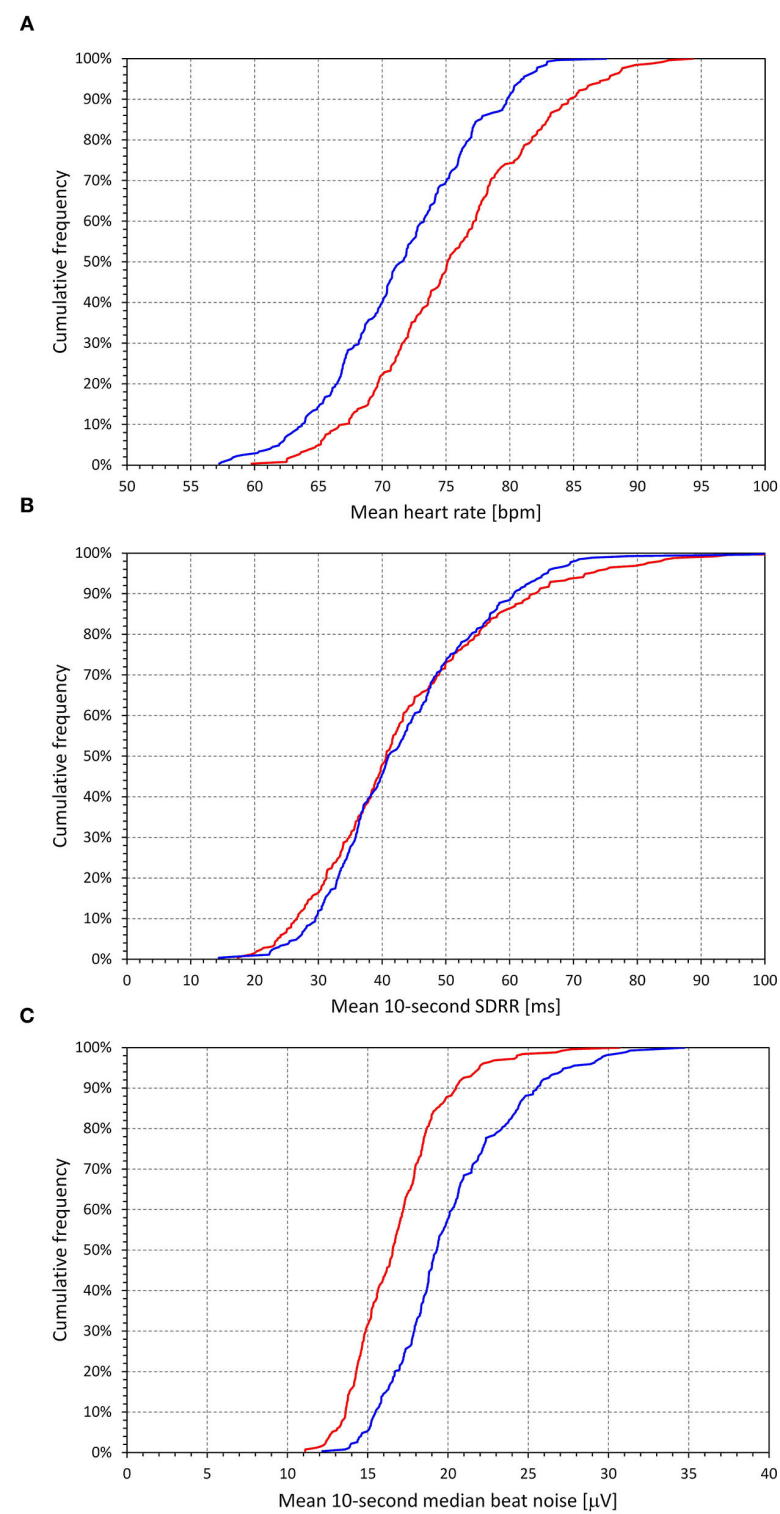
Population distributions of principal ECG measurements (intra-subject means of measurements obtained in individual 10-s ECG segments accepted in the recordings of the given subject). The top **(A)**, middle **(B)**, and bottom **(C)** show the distributions of mean heart rates, mean values of SDRR, and mean values of 10-s median beat noise, respectively. In each panel, the red and blue lines show the distributions among females and males, respectively.

As expected (see Figure 6), the intra-subject mean heart rate of the analyzed 10-s ECG samples was faster in females (75.6 ± 6.8 beats per minute — bpm) than in males (71.5 ± 6.0 bpm, *p* < 0.0001). The mean 10-s SDRR was not different between sexes (43.7 ± 15.4 and 43.5 ± 12.8 ms in females and males, respectively). Somewhat unexpectedly, the intra-subject means of 10-s median beat noise were smaller in females (16.8 ± 3.0 μV) than in males (20.1 ± 3.9 μV, *p* < 0.0001). Despite the heart rate differences, the mean duration of the uncorrected QT interval was longer in females (386.9 ± 18.4 ms) than in males (379.5 ± 16.0 ms, *p* < 0.0001).

#### Relationship to Age

**Figure 7:**
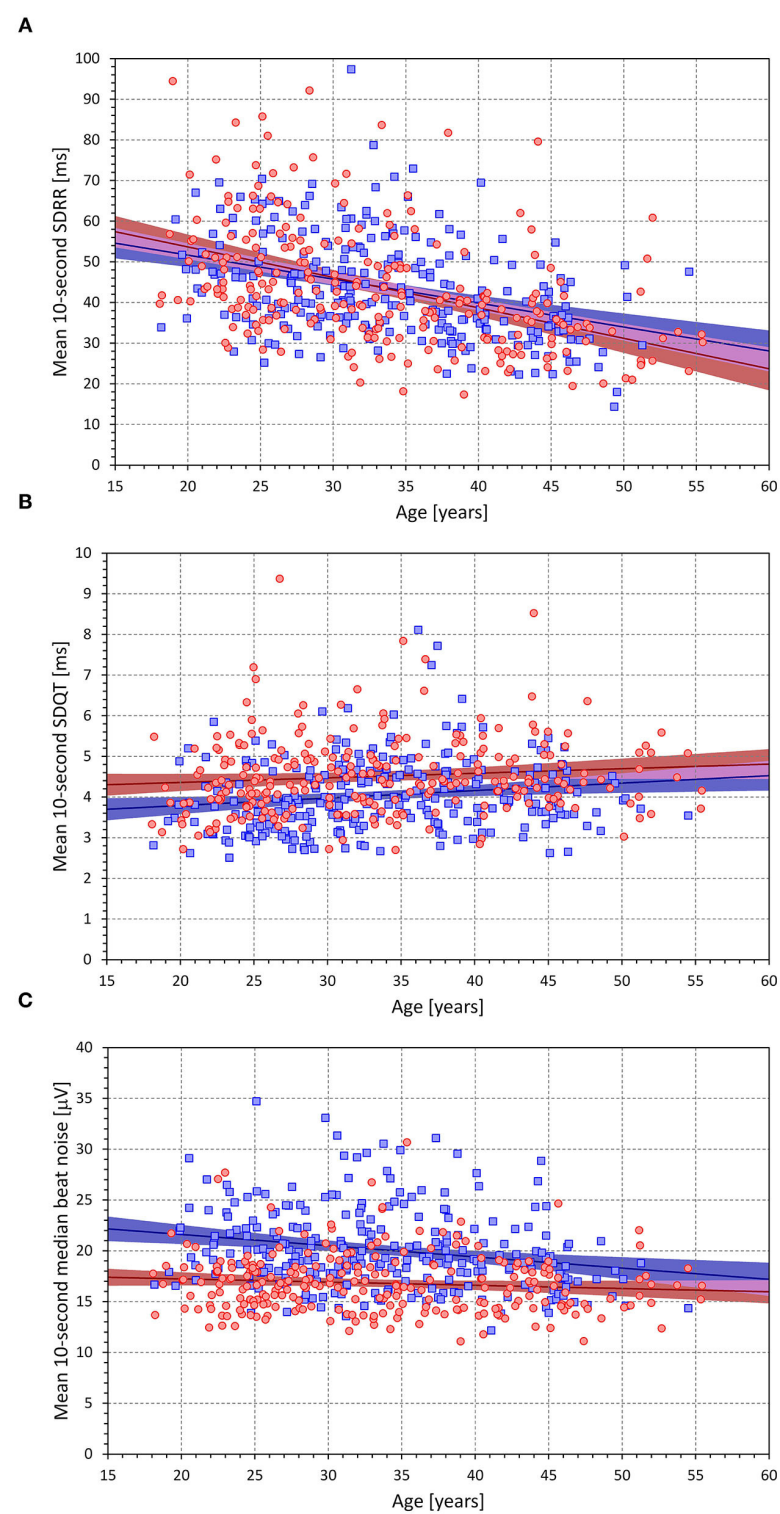
The figure shows age influence on intra-subject means of 10-s SDRR [upper **(A)**], 10-s SDQT [middle **(B)**], and 10-s median beat noise [bottom **(C)**]. In each panel, the red circle and blue square marks correspond to females and males, respectively. The red and blue lines show the linear regression models between the displayed characteristics and age, the light colored red and blue bands show the 95% confidence intervals of the linear regressions, the light-colored violet areas show the overlaps between the regression confidence intervals of both sexes.

As again expected (see Figure 7), intra-subject mean SDRR was significantly decreasing with increasing age (*p* < 0.0001 in both sexes). The intra-subject means of 10-s median beat noise were significantly decreasing with age in males (*p* = 0.0003) but were only non-significantly decreasing with advancing age in females. The age-related increase in mean 10-s SDQT was significant in males (*p* = 0.007) but was only statistically borderline in females (*p* = 0.092).

#### Correlations

**Figure 8:**
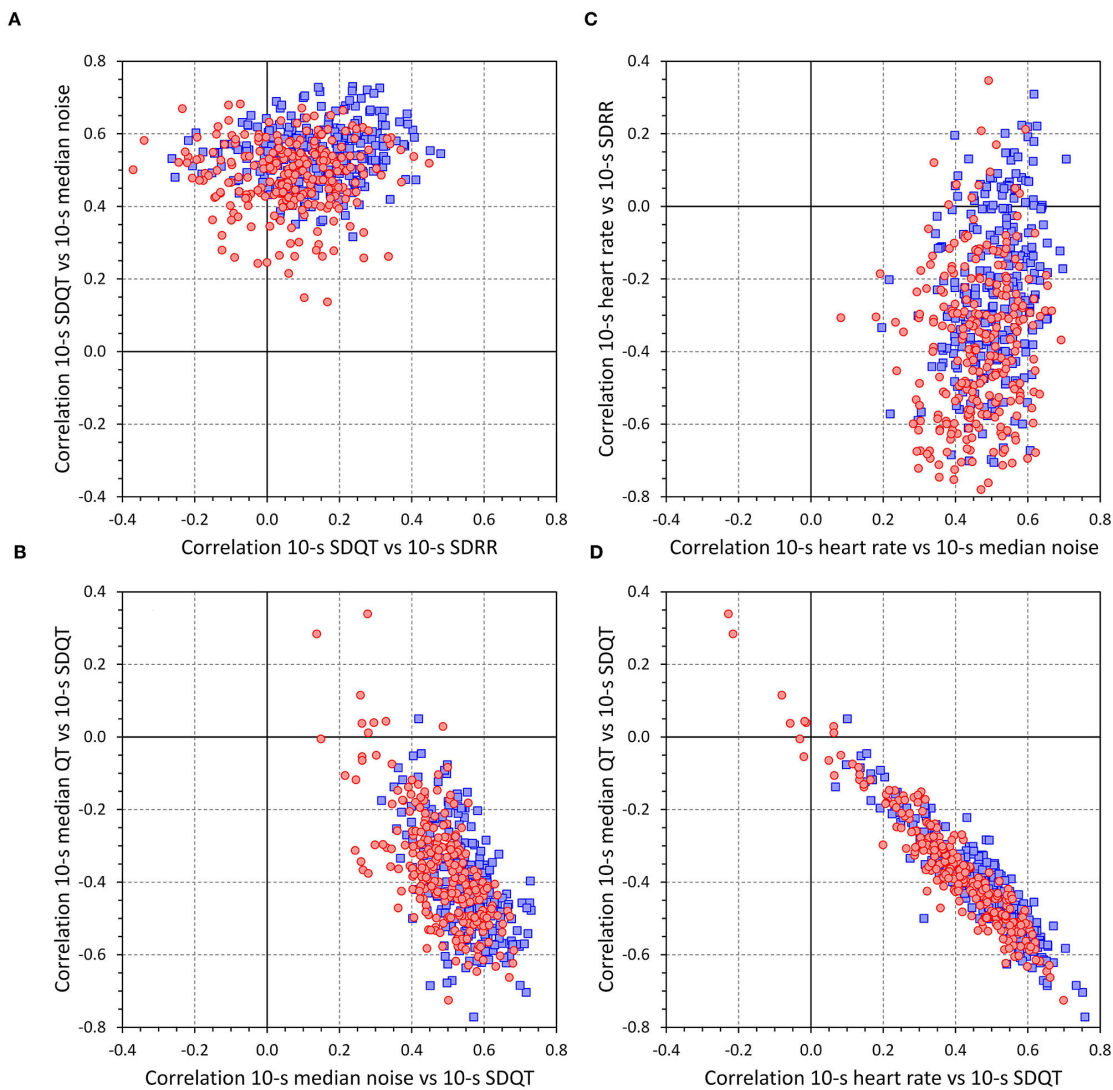
The panels of the figure show scatter diagrams between intra-subject correlations between different measurements in accepted 10-s ECG segments [see the labels of the axes of **(A–D)** for the specification of the correlations]. In each panel, red circles and blue squares correspond to females and males, respectively.

The intra-subject correlations between the 10-s ECG measurements are graphically summarized in Figure 8. Similar to the individual beat measurements, the correlations between SDQT and SDRR were modest, 0.073 (−0.016–0.158) in females and 0.146 (0.057–0.235) in males (sex comparison *p* < 0.0001). The correlations between SDQT and 10-s median beat noise were substantially stronger, 0.498 (0.432–0.549) in females and 0.549 (0.497–0.596) in males (*p* < 0.0001). The correlations between 10-s heart rate and 10-s median beat noise were 0.464 (0.398–0.538) in females and 0.511 (0.444–0.569) in males *(p* < 0.0001) and influenced the correlations between SDQT and 10-s heart rate which were 0.413 (0.323–0.515) in females and 0.492 (0.392–0.565) in males (*p* < 0.0001). These were very similar (albeit with opposite signs) to the correlations between SDQT and 10-s median QT interval, −0.390 (−0.490–−0.296) on females and −0.427 (−0.504–−0.329) in males (*p* = 0.0016). As expected, the correlations between 10-s heart rate and SDRR were mostly (but not exclusively) negative, −0.387 (−0.548–−0.241) in females and −0.246 (−0.397–−0.073) in males (*p* < 0.0001).

**Figure 9:**
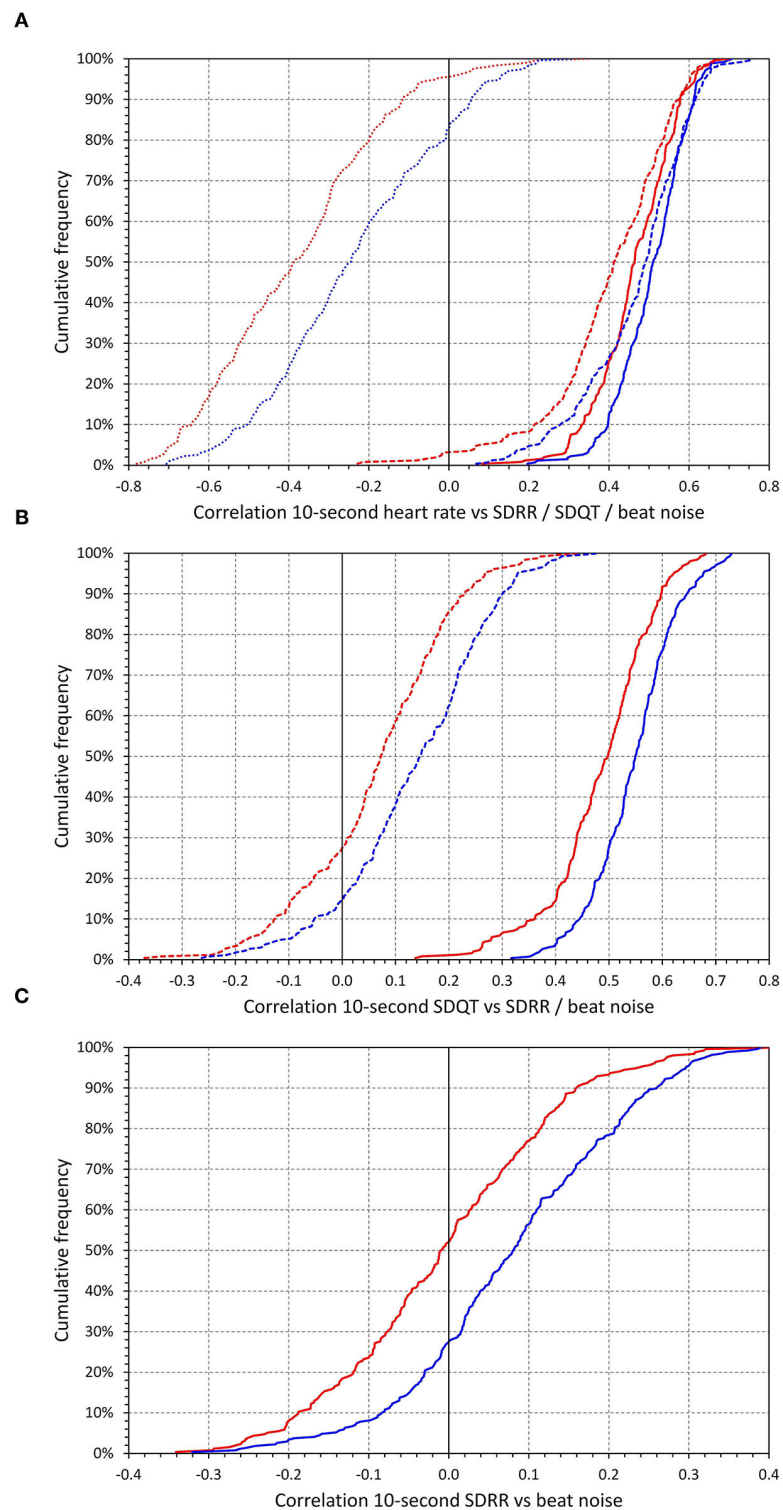
The top **(A)** shows cumulative distributions of intra-subject correlations between 10-s heart rate and 10-s median beat noise (solid lines), intra-subject correlations between 10-s heart rate and 10-s SDQT (dashed lines), and intra-subject correlations between 10-s heart rate and 10-s SDRR (dotted lines). The middle **(B)** shows cumulative distributions of intra-subject correlations between 10-s SDQT and 10-s median beat noise (solid lines) and intra-subject correlations between 10-s SDQT and 10-s SDRR (dashed lines). The bottom **(C)** shows cumulative distributions of intra-subject correlations between 10-s SDRR and 10-s median beat noise. In each panel, the red and blue lines show the distributions in females and males, respectively.

Cumulative distributions of the data presented in Figure 8 are shown in Figure 9.

**Figure 10:**
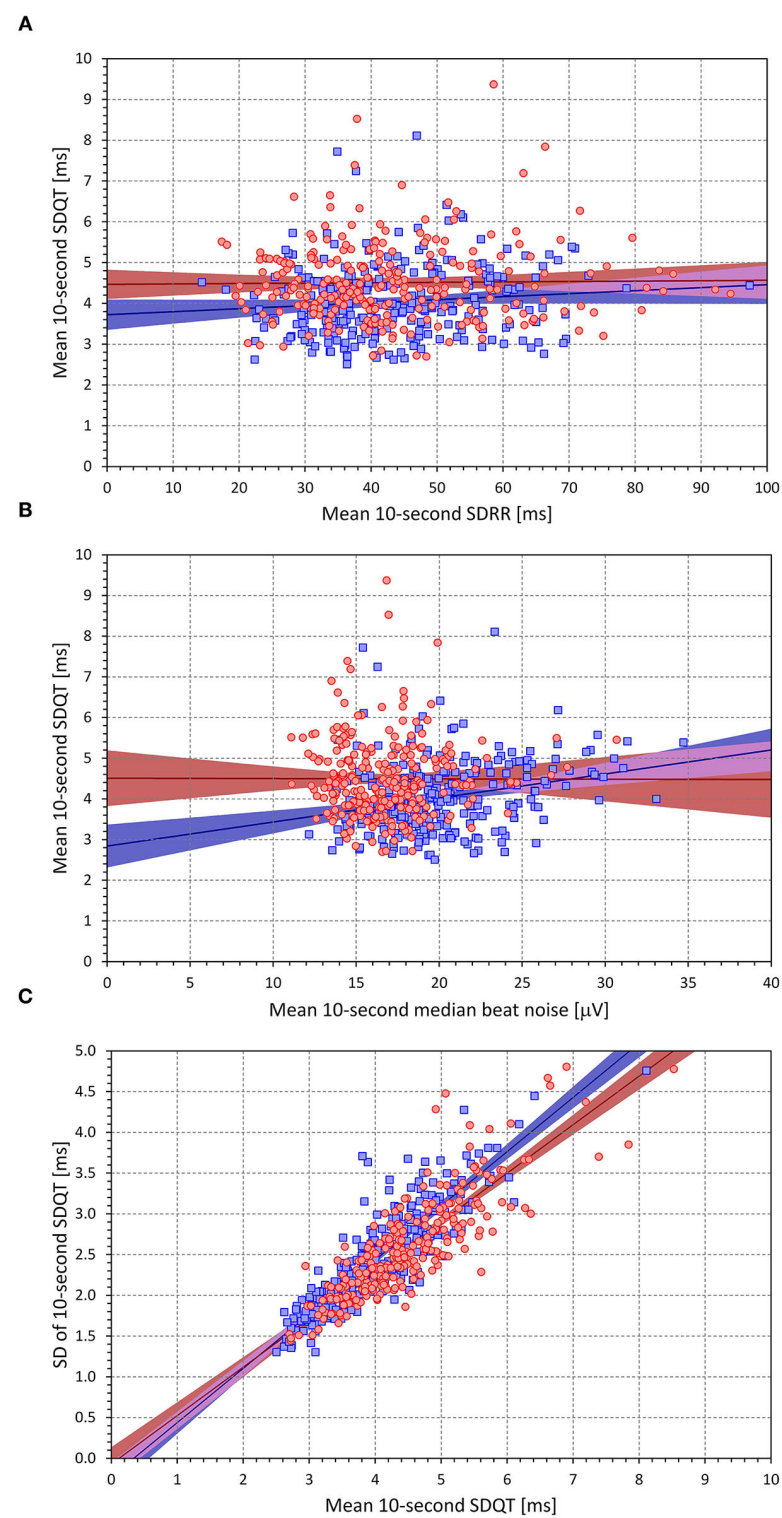
Scatter diagrams showing population relationship between intra-subject means of 10-s SDRR and 10-s SDQT [top **(A)**], intra-subject means of 10-s median beat noise, and 10-s SDQT [middle **(B)**], and intra-subject means of 10-s SDQT and intra-subject standard deviations of 10-s SDQT [bottom **(C)**]. In each panel, the red circle and blue square marks correspond to females and males, respectively. The red and blue lines show the linear regression models between the displayed characteristics, the light colored red and blue bands show the 95% confidence intervals of the linear regressions, the light-colored violet areas show the overlaps between the regression confidence intervals of both sexes.

Contrary to these intra-subject correlations, some of the inter-subject correlations between the intra-subject mean values were very shallow (Figure 10). The intra-subject means of SDQT were not related to the intra-subject means of SDRR; the intra-subject means of SDQT were related to means of 10-s median noise in males (*p* < 0.0001) but not in females.

As expected, the intra-subject means of SDQT were very strongly related to the intra-subject variability of SDQT.

#### Regression-Based Corrections of SDQT

**Figure 11:**
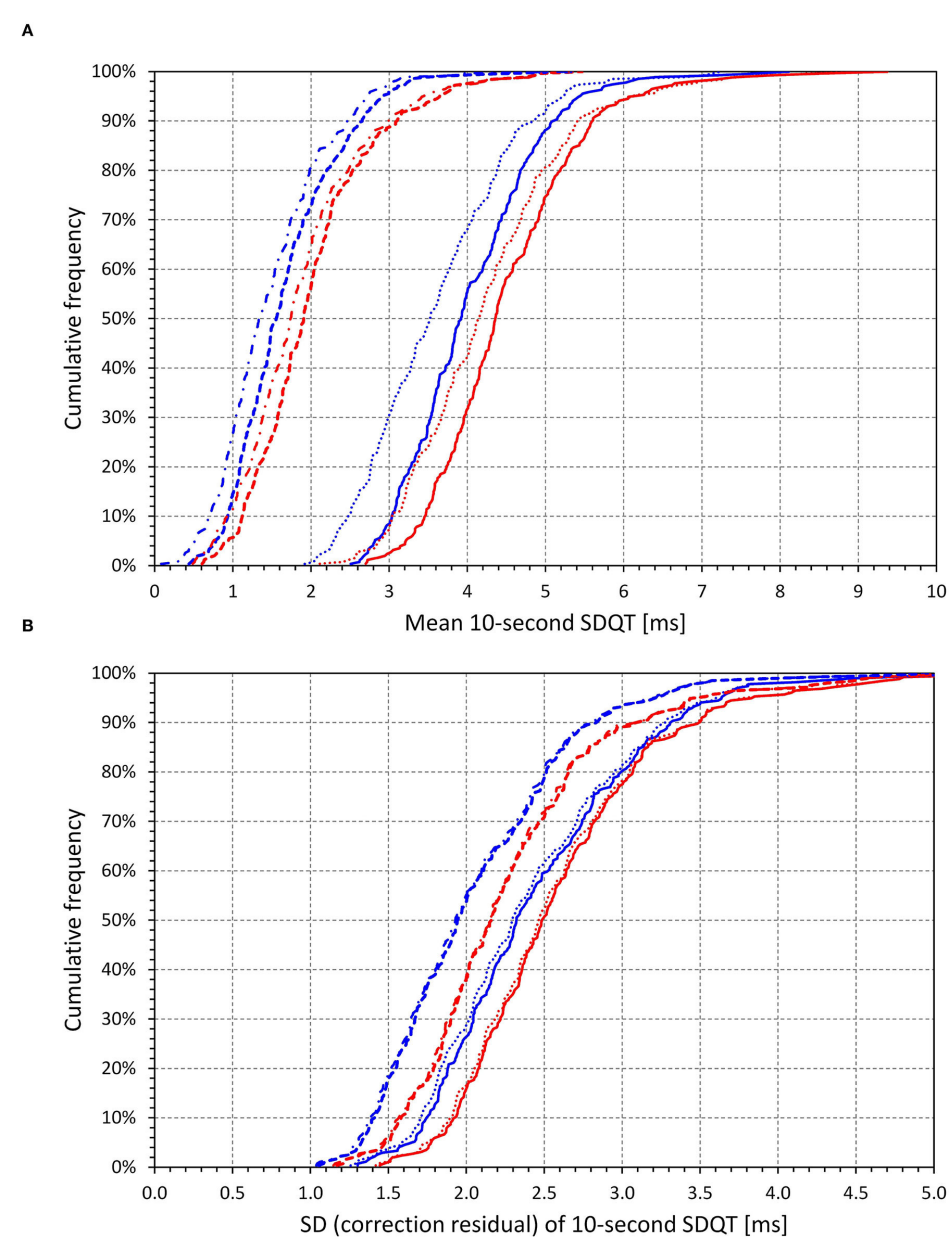
Cumulative distributions of intra-subject means of 10-s SDQT [top **(A)**] and of intra-subject standard deviations of the 10-s SDQT [bottom **(B)**]. The full lines show the distribution of source data without any regression correction, the dotted lines regression-corrected SDQT values for SDRR [top **(A)**] and residuals of linear regression of SDQT to SDRR [bottom **(B)**], the bold dashed lines regression-corrected SDQT values for 10-s median beat noise [top **(A)**] and residuals of linear regression of SDQT to 10-s median beat noise [bottom **(B)**], and dashed and dotted lines regression-corrected SDQT values for a combination of SDRR and 10-s median beat noise [top **(A)**] and residuals of linear regression of SDQT to a combination of SDRR and 10-s median beat noise [bottom **(B)**]. The red and blue lines in both panes correspond to females and males, respectively.

Figure 11 shows that the intra-subject mean SDQT values were significantly larger in females (4.51 ± 0.96 ms) than in males (4.05 ± 0.88 ms, *p* < 0.0001). The intra-subject variabilities, that is standard deviations of SDQT, were also larger in females (2.61 ± 0.67 ms) than in males (2.46 ± 0.66 ms, *p* = 0.01).

**Figure 12:**
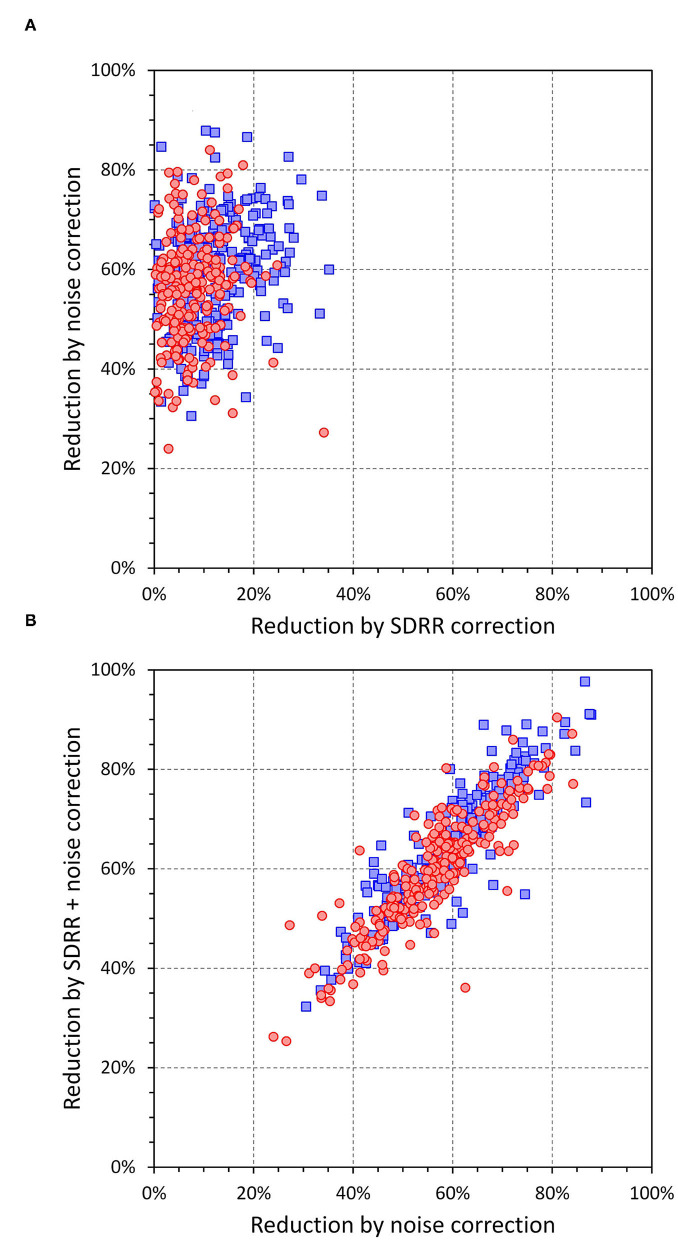
The reduction values shown in this figure are defined as values of relative decrease of intra-subject regression corrected central SDQT values calculated as a proportion of uncorrected intra-subject means of SDQT. The top **(A)** shows a scatter diagram of the reductions involving regression to SDRR vs. those involving 10-s median beat noise; the bottom **(B)** shows a scatter diagram of the reductions involving 10-s median beat noise vs. those involving regression to a combination of SDRR and 10-s median beat noise. In both panels, red circles and blue squares correspond to female and males, respectively.

**Figure 13:**
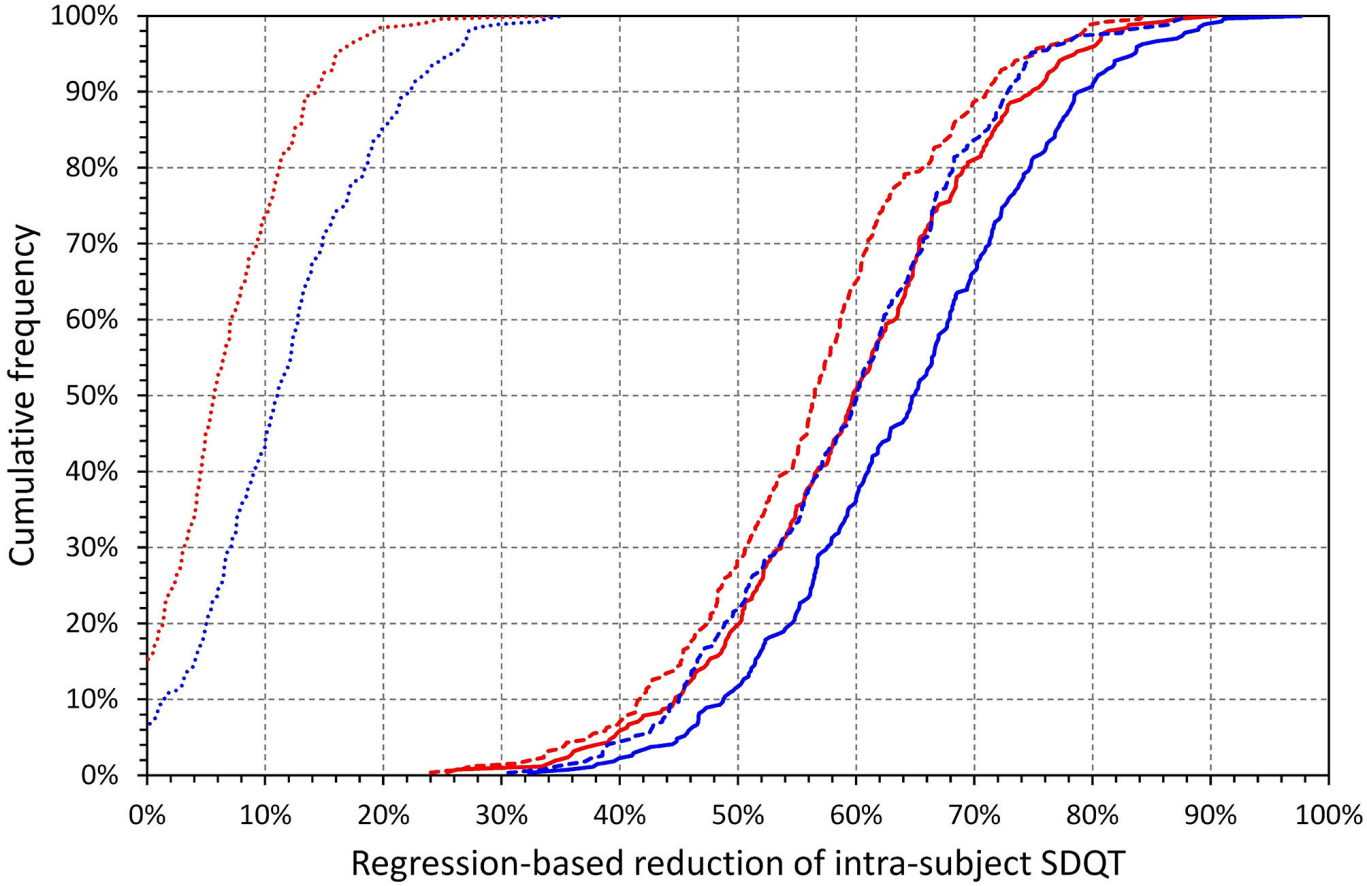
Cumulative distributions of relative decreases of intra-subject regression corrected central SDQT values calculated as a proportion of uncorrected intra-subject means of SDQT (see the section on Statistics and data presentation for details). The reductions by regression correction by SDRR are shown in dotted lines, the reduction by 10-s median beat noise in dashed lines, and the reduction by a regression combination of both SDRR and 10-s median beat noise by solid lines. The red and blue lines correspond to females and males, respectively.

Figure 11 further shows that regression-based correction of intra-subject SDQT values for the underlying SDRR values reduces SDQT (median, IQR) by only 5.7% (2–10.3%) in females and 11.1% (6.2–16.8%) in males. On the contrary, the correction for the underlying 10-s median noise reduces SDQT by 56.5% (48.7–62.3%) in females and by 60.1% (50.9–66.6%) in males. Additional combined correction for both the underlying SDRR and 10-s median noise reduces SDQT only a little bit more by 59.7% (51.8–66.8%) in females and by 65.0% (56.3–72.6%) in males. All these relative corrections show significant sex differences (p between <0.0001 and 0.001). The comparisons of these corrections are shown in Figures 12, 13.

Figure 11 also shows a reduction of the intra-subject variability of SDQT. The reduction by correction for underlying SDRR values is only minimal, by 0.54% (0.10–1.32%) in females and by 1.33% (0.46–2.68%) in males. The correction for the underlying 10-s median noise led to a larger reduction in the intra-subject SDQT variability by 13.3% (9.5–16.7%) in females and by 16.5% (13.1–19.7%) in males. Combined correction for both the underlying SDRR and 10-s median noise reduced SDQT variability only a tiny bit more by 13.7% (10.3–17.4%) in females and by 17.4% (13.7–20.7%) in males. As with the corrections of the SDQT values, all these corrections of SDQT variability show significant sex differences (all *p* < 0.0001).

**Figure 14:**
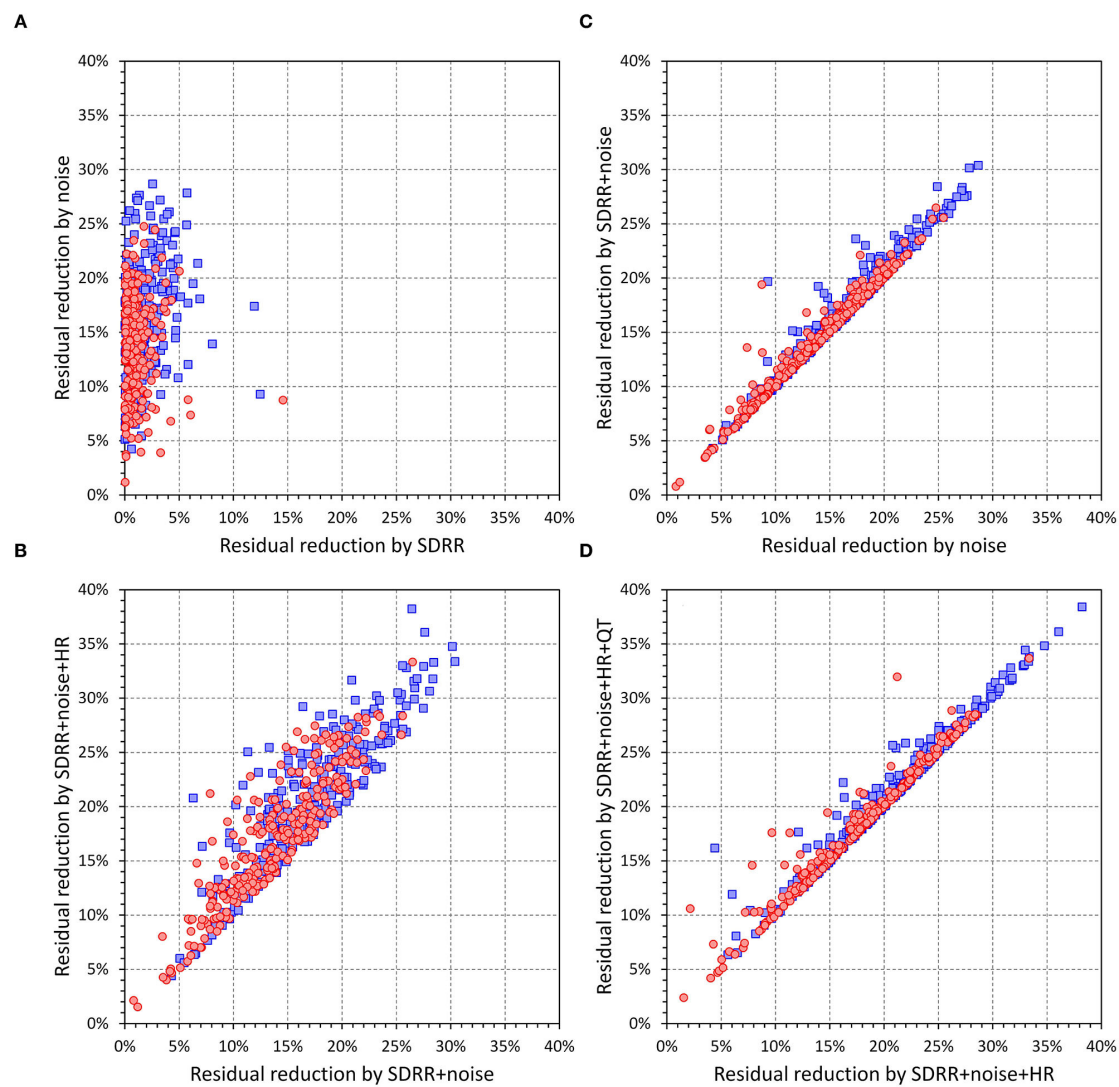
The residual reductions shown in this figure **(A–D)** are defined as values of relative decrease of intra-subject residuals of linear regressions SDQT calculated as proportions of intra-subject standard deviations of SDQT. Axis labels of SDRR, noise, SDRR + noise, SDRR + noise + HR, and SDRR + noise + HR + QT designate intra-subject residuals of SDQT vs. SDRR; 10-s median beat noise; combination of SDRR and 10-s median beat noise; the combination of SDRR, 10-s median beat noise, and 10-s heart rate; and combination of SDRR, 10-s median beat noise, 10-s heart rate, and 10-s median QT interval duration, respectively. In all panels, red circles and blue squares correspond to females and males, respectively.

**Figure 15:**
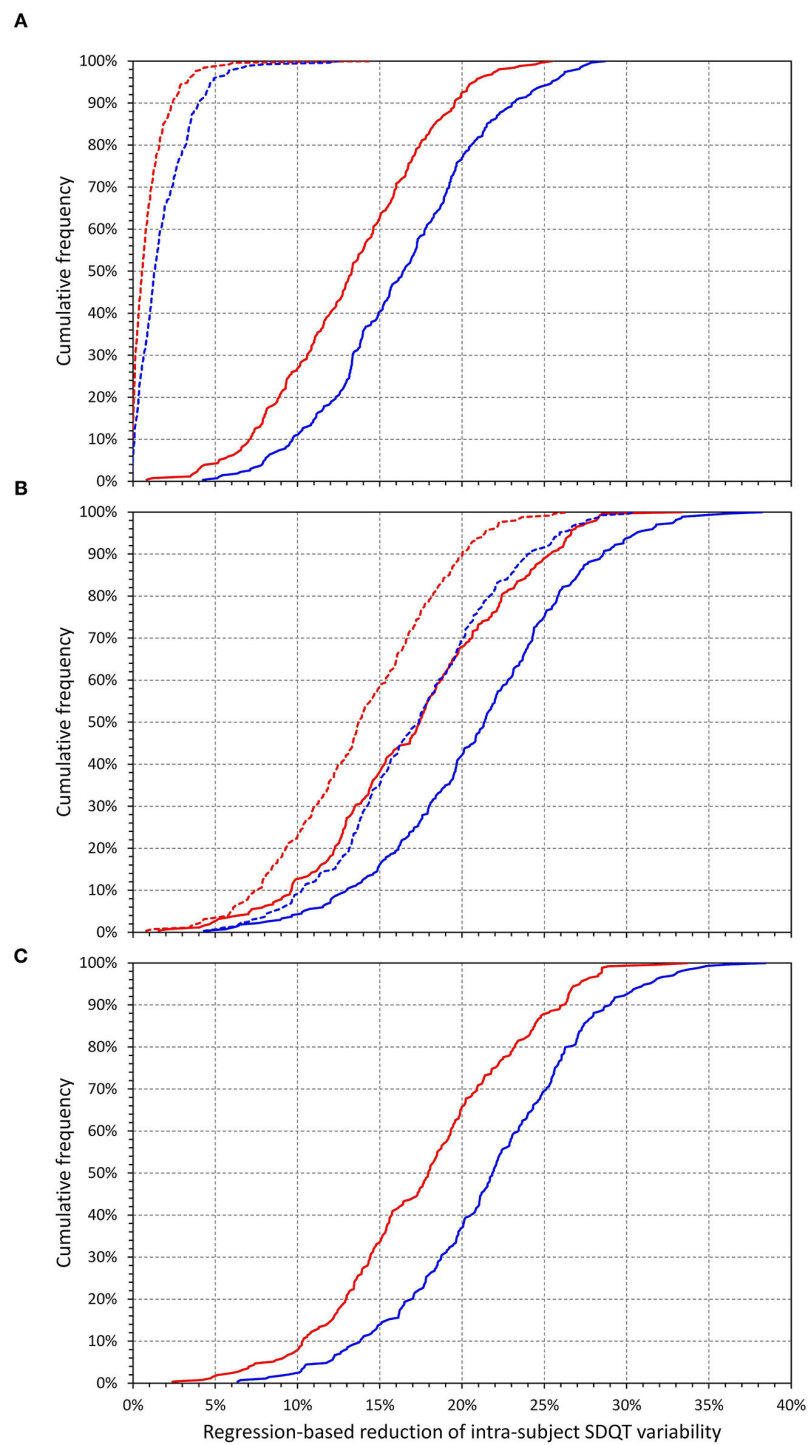
Cumulative distributions of the relative decrease of intra-subject residuals of linear regressions SDQT calculated as proportions of intra-subject standard deviations of SDQT (see the section on Statistics and data presentation for details). The top **(A)** shows the reductions by 10-s median beat noise correction (solid lines) and by SDRR correction (dashed lines). The middle **(B)** shows reductions by a correction for a combination of 10-s median beat noise and SDRR (dashed lines) and for a combination of 10-s median beat noise, SDRR, and 10-s heart rate (solid lines). The bottom **(C)** shows reductions by a correction for a combination of 10-s median beat noise, SDRR, 10-s heart rate, and 10-s median QT interval duration. In each panel, the red and blue lines show the distributions among females and males, respectively.

Figures 14, 15 show the comparison of these SDQT variability reductions and also show that additional multi-variable regression involving also underlying 10-s heart rate and the 10-s median QT interval duration led only to further marginal corrections of SDQT variability.

## Discussion

The study leads to two principal observations that are of importance for both physiologic understandings of cardiac repolarisation variability and practical interpretation of ECG measurements. Firstly, our observations contradict the concept of systematic and reproducible immediate RR interval effect on QT interval duration. Secondly, the data analyses suggest that the standard approaches to the assessment of beat-to-beat QT interval variability might overestimate true QT variability if the noise within the source ECG recordings is not carefully controlled or if the QT variability is not corrected for the extent of biological and technical noise.

As seen in the examples in Figures 1, 2, as well as in the analysis of individual beat data, the relationship between ΔQT and ΔRR was not only shallow but also largely non-reproducible. Indeed, the linear relation of QT interval changes to RR interval changes accounted only for a small number of single percentages of the intra-subject beat-to-beat ΔQT variability. This non-systematic relationship between QT interval changes and RR interval changes was further confirmed by the remarkably low intra-subject correlations between SDQT and SDRR (see Figure 7). Hence this study is very much in agreement with a number of previous studies that challenged that concept of immediate RR interval effect on the QT interval. While beat-to-beat RR interval prolongation and shortening is more frequently followed by QT interval prolongation and shortening than by the opposite QT interval changes, the opposite changes appear in approximately one-third of the beats (as seen by the analysis of concordant and discordant beat frequency as shown in Figure 4).

This challenge to the concept of immediate RR interval effect has two practical implications. Firstly, as repeatedly confirmed in previous publications, QT intervals duration should not be corrected for the preceding RR interval, and the complete history of preceding heart rate should be taken into account (Franz et al., 1988; Lau et al., 1988; Malik et al., 2004, 2008b, 2016; Jacquemet et al., 2014; Gravel et al., 2017). Indeed, the intra-subject slopes of ΔQT/ΔRR linear regressions were approximately one-tenth of the QT/RR regressions used in population-based QTc corrections (Sagie et al., 1992). This is in good agreement with the previously established profiles of QT/RR hysteresis. While the need of considering the longer heart rate history of any QT interval measurement is now largely accepted and incorporated into the design of studies investigating heart rate corrected QT interval, it is regrettable when statements that “QT and RR value for each beat will be used for heart rate correction” are written in rather recent analysis plans of laboratories providing ECG services for pharmaceutical industry (ERT, 2020). Secondly, the lack of immediate RR interval effect has implications for the physiologic interpretation of the QT variability index (Berger et al., 1997; Baumert et al., 2016). As we have previously discussed (Andršová et al., 2020), increases in the QT variability index might be caused both by increases in beat-to-beat QT variability and by decreases in heart rate variability which the index cannot distinguish (Tereshchenko et al., 2012). It can only be suggested that in future studies of diagnostic and prognostic implications of QT variability, separate measurements of QT variability and RR variability are investigated separately so that the implications of increased QT variability are not confused with heart rate variability reduction.

The immediate RR interval effect needs to be distinguished from general QT interval dependency on the underlying heart rate which is indisputable. Dependent on the degree of sinus arrhythmia, a single RR interval measurement offers a more or a less imprecise estimate of underlying heart rate. It is therefore not surprising that repeated studies have found some dependency of QT interval, QTpeak interval, and of other repolarisation characteristics on the preceding RR interval (Funck-Brentano and Jaillon, 1993; Porta et al., 1998a). However, the concept of immediate RR interval effect postulates something different since it proposes that beat-to-beat shortening and prolongation of RR interval leads to consistent shortening and prolongation of the immediately following QT interval. This concept is clearly invalidated by this study.

Our finding of the association of beat-to-beat QT variability with the morphological instability (which, for simplicity's sake, we call “noise”) of the source ECG tracing should not be interpreted as a suggestion that there are no beat-to-beat QT changes and that only morphology changes influence the beat-to-beat measurements. Morphological ECG changes might also have valid biological basis [e.g., related to respiration (Noriega et al., 2012; Sadiq et al., 2021), posture (Markendorf et al., 2018), food intake (Täubel et al., 2019), mental stress (Hwang et al., 2018), and other external inputs and physiologic reflexes]. While some of these mechanisms might not be fast enough to contribute beat-to-beat changes in QT interval measurements, it seems clear that the measurement of the differences between individual beats and the global representative waveform is not necessarily reflecting only technical ECG imperfection. Still, the association of short-term QT variability with the “noise” measurements was so surprisingly strong, that we are of the opinion that some truly technical noise levels were involved and their influence on the beat-to-beat QT interval measurement needs to be considered. This is in good agreement with the old observations that the so-called QT dispersion was highly contributed by ECG signal imperfections (Kautzner et al., 1994; Kors and van Herpen, 1998; Rautaharju, 1999; Malik and Batchvarov, 2000). Our observations were made despite the careful selection of only beats with very high correlations between the individual beat morphologies and the representative waveforms (i.e., despite using only good quality recordings). Therefore, it seems appropriate to suggest that in future studies of beat-to-beat QT variability, the objective assessment of ECG noise contents is incorporated (Batchvarov et al., 2002; Lee et al., 2012; Abreu et al., 2017; Everss-Villalba et al., 2017) so that it can be shown that any reported QT variability changes are independent of ECG noise pollution.

Two further comments are important for the practical implications of our findings of the influence of signal variability which we, for simplicity, call the noise. Firstly, the noise levels that we have observed were only tiny (see the bottom panel of Figure 6) in comparison to what would be called noise-polluted ECG in clinical and/or experimental practice as well as tiny in comparison to technical studies that previously investigated the effects of ECG noise on the ECG measurement and diagnostic accuracy (Porta et al., 1998b; Chang, 2010; Li et al., 2014; Tayel et al., 2018). Indeed, the median noise levels well below 20 μV (see Figure 6) would be practically invisible on standard ECG prints. Secondly, and perhaps more importantly, our observation of the noise influence calls for careful interpretations of data obtained by the simple correlation matching algorithm (Berger et al., 1997; Baumert et al., 2016) that has been the basis for many if not the majority of previous studies of beat-to-beat QT variability. Regardless of whether the signal morphological variability is caused by biological or technical factors, it is not surprising that even tiny morphological changes influence the correlation matching algorithm. Hence, comparisons of beat-to-beat QT variability between different data sets might be problematic (if not entirely misleading) unless the QT variability data are corrected for the underlying morphological variability. Establishing an algorithm that would not only allow beat-to-beat QT measurements but would also produce results independent of measurable morphological variability remains a challenge.

The concept of correction of the SDQT values replicated the intra-subject regressions-based corrections of QT, JT, and JTp intervals for the underlying heart rate (Garnett et al., 2012; Panicker et al., 2018; Hnatkova et al., 2019). Since the intra-subject spreads of the data were rather wide (see examples in Figures 1, 2) we have not attempted to use any curvilinear regressions (Malik et al., 2012b) and used linear regression as the least biased approach. Contrary to the QT heart rate correction that, by the usual definition, estimates the duration of the QT interval at RR interval of 1 s, we used corrections estimating the SDQT values at the zero level of SDRR and/or zero level of ECG morphological variability. Possibly, similar corrections might be used in future studies of QT variability if, for some reason, multivariable regressions also involving heart rate variability and ECG signal-to-noise ratio do not appear appropriate.

It is not surprising that we found increased ECG morphological variability during faster heart rates (see Figure 7). It is thus possible that previous observations of increased QT variability at faster heart rates (Hnatkova et al., 2013) were influenced by measurement bias. The relationship between heart rate and QT variability also explains the somewhat counterintuitive observation of the negative correlation between QT interval duration and QT variability (see Figure 7). As heart rate increases, uncorrected QT interval decreases which leads to this somewhat unexpected observation. Since it has been observed that faster heart rates lead to increased morphological variability, it remains challenging to investigate whether short-term SDQT is influenced by autonomic conditions independently of the autonomic influence on SDQT measurement accuracy.

### Limitations

The limitations of our study also need to be considered. While a number of previous QT variability studies used longer ECGs, we analyzed 10-s ECG segments since these are more relevant for practical purposes. We are unable to comment on whether the very same observations would be obtained with longer recordings. Nevertheless, since every longer ECG recording is, in principle, a series of shorter ECG segments, it is unlikely that with longer recordings, our observations would be principally different. The investigated population included neither very young nor very old subjects. The investigations of the relationship to age were therefore limited to the available age ranges. Finally, since the study data were obtained from clinical pharmacology investigations in healthy subjects, we are unable to comment on whether the same results would have been found if researching populations with clinically well-defined pathological characteristics. Nevertheless, our observations on the influence of recording quality and morphological instability still have implications for clinical investigations since anecdotal experience from clinical investigations suggests that poorer ECG quality is related to worsened outcomes.

## Conclusion

Despite these limitations, the study shows that the concept of immediate RR interval effect on the duration of subsequent QT interval duration is questionable since the beat-to-beat QT interval variability is little dependent on the underlying RR interval variability. Importantly, the analyzed data provide substantial evidence that even if only stable beat-to-beat measurements of QT interval are used, the QT interval variability is still substantially influenced by morphological variability and noise pollution of the source ECG recordings. The quality and signal-to-noise ratio thus needs to be carefully considered in future studies of QT interval variability.

## Data Availability Statement

The raw data supporting the conclusions of this article will be made available by the authors, without undue reservation but pending the approval by the sponsors of the source clinical studies.

## Ethics Statement

The studies involving human participants were reviewed and approved by Parexel in Baltimore, California Clinical Trials in Glendale, and Spaulding in Milwaukee. The patients/participants provided their written informed consent to participate in the source clinical studies.

## Author Contributions

IA, KH, MM, and OT: study design and initial manuscript draft. KH and MM: software development and statistics and figures. GS, IA, KMH, MŠ, OT, PB, PS, and TN: ECG interpretation and ECG measurement. GS, TN, OT, and PS: supervision of the measurements. GS, MM, and TN: quality control of the measurements. All authors approved the final manuscript of the submission.

## Funding

This work was supported in part by the British Heart Foundation New Horizons Grant NH/16/2/32499, by Ministry of Health, Czech Republic, Conceptual Development of Research Organization (Grant FNBr/65269705), and by the Specific Research of Masaryk University MUNI/A/1450/2021.

## Conflict of Interest

The authors declare that the research was conducted in the absence of any commercial or financial relationships that could be construed as a potential conflict of interest.

## Publisher's Note

All claims expressed in this article are solely those of the authors and do not necessarily represent those of their affiliated organizations, or those of the publisher, the editors and the reviewers. Any product that may be evaluated in this article, or claim that may be made by its manufacturer, is not guaranteed or endorsed by the publisher.
